# The association between perceived hospital ethical climate and self-evaluated care quality for COVID-19 patients: the mediating role of ethical sensitivity among Chinese anti-pandemic nurses

**DOI:** 10.1186/s12910-021-00713-4

**Published:** 2021-10-27

**Authors:** Wenjing Jiang, Xing’e Zhao, Jia Jiang, Huilin Zhang, Shujuan Sun, Xianhong Li

**Affiliations:** 1 Xiangya School of Nursing, Central South University, 172 Tong Zi Po Road, Changsha, 410013 Hunan China; 2 Department of Nursing, Zigong First People’s Hospital, Zigong, 643000 China; 3 Department of Liver Transplantation, The Second Xiangya Hospital of Central South University, Changsha, 410011 China; 4 West China School of Pharmacy, Sichuan University, Chengdu, 610041 China; 5 Department of Nursing, The Second Xiangya Hospital of Central South University, Changsha, 410011 China; 6 Department of Gynecology, The Second Xiangya Hospital of Central South University, Changsha, 410011 China

**Keywords:** COVID-19, Hospital ethical climate, Ethical sensitivity, Care quality, Mediation effect

## Abstract

**Background:**

The COVID-19 pandemic called for a new ethical climate in the designated hospitals and imposed challenges on care quality for anti-pandemic nurses. Less was known about whether hospital ethical climate and nurses’ ethical sensitivity were associated with care quality. This study examined the association between the perceived hospital ethical climate and self-evaluated quality of care for COVID-19 patients among anti-pandemic nurses, and explored the mediating role of ethical sensitivity in this relationship.

**Methods:**

A cross-sectional study was conducted through an online survey. A total of 399 anti-pandemic nurses from ten designated hospitals in three provinces of China were recruited to fill out an online survey. Multiple linear regression analysis and a bootstrap test were used to examine the relationships between ethical climate, ethical sensitivity and care quality.

**Results:**

Nurses reported mean scores of 4.43 ± 0.577 (out of 5) for hospital ethical climate, 45.00 ± 7.085 (out of 54) for ethical sensitivity, and 5.35 ± 0.661 (out of 6) for self-evaluated care quality. After controlling for covariates, perceived hospital ethical climate was positively associated with self-evaluated care quality (direct effect = 0.710, 95% confidence interval [CI] 0.628, 0.792), and was partly mediated by ethical sensitivity (indirect effect = 0.078, 95% confidence interval [CI] 0.002, 0.145).

**Conclusions:**

Chinese nurses who cared for COVID-19 patients perceived high levels of hospital ethical climate, ethical sensitivity, and self-evaluated care quality. Positive perceptions of hospital ethical climate were both directly associated with a higher level of self-evaluated care quality and indirectly associated, through the mediation effect of ethical sensitivity among anti-pandemic nurses.

## Background

After the first case of COVID-19 was reported in December 2019, the outbreak gradually spread nationwide, becoming an epidemic that subsequently spread throughout the world, reaching pandemic proportions [1]. According to the State Council Information Office ofthe People’s Republic of China [2], the epidemic is being managed effectively across the country. However, during the initial outbreak, frontline nurses (usually known as anti-pandemic nurses) faced an unprecedented workload with limited spaces to hold patients and without enough protective equipment. They were required not only to save lives but also to protect their own [3, 4]. By February 24, 2020, a total of 3,387 Chinese healthcare workers were infected with COVID-19 in 476 medical institutions (2,055 confirmed cases, 1,070 clinically diagnosed cases, and 157 suspected cases) [5]. All along, frontline care was considered as an “ethically laden practice” [6]. Nurses were responsible for patient care in a context where they were uncertain of their competency to treat a novel infection and were facing new occupational infection risk. It was reported that a considerable proportion of anti-pandemic nurses experienced symptoms of depression, anxiety, insomnia, and distress [7].

Ethical climate refers to “individual perceptions of the organization that influence attitudes and behavior and serve as a frame of reference for employee behavior” [8]. As researchers in several countries paid more attention to the ethical climate in medical settings, the concept of hospital ethical climate has been gradually clarified. The hospital ethical climate is usually defined as nurses’perceptions on how ethical issues are handled in their work environment [9]. It is assessed by nurses’ perceptions of how satisfied they are with their interactions with peers, patients, managers, and physicians, and how the hospital’s vision and goals fit their professional values [8, 9]. Therefore, the hospital ethical climate perceived by nurses was undoubtedly influenced by organizational factors such as the culture, values and policies of the hospitals they work in. Additionally, the strength of a hospital’s ethical climate might affect nurses’ attitudes about ethical issues and ethical decision-making. According to international literature [10–12], ethical climate plays a crucial role in determining nurses’ job satisfaction and nursing care behaviors, which might further affect patient care.

During the early stage of the outbreak, the designated hospitals in Wuhan tried to establish a sound ethical climate in order to provide good quality of care to patients and stop the epidemic. First, Hospital management emphasized the meaningfulness of participating in frontline care. Hospital management pointed out the global acknowledgment of the essential role that nurses have played in disease prevention, health promotion, patient education, and care of sick and wounded patients. As suggested by Florence Nightingale, “to heal the wounded and rescue the dying” has always been considered to be the duty of nurses [13]. Thus, many nurses in other regions of China volunteered to come to Hubei to offer frontline care. Up to March 1, more than 42,000 healthcare workers, including 28,600 nurses from other provinces in China, arrived in Hubei Province to assist in treating patients [14]. Furthermore, the media portrayed many nurses, doctors, and other healthcare workers as heroes, supermen and -women, warriors, or martyrs for their care of COVID-19 patients, and the public admired and praised them [15]. This reinforced the healthcare workers’ perception of their professional duties.

Second, emphasizing the importance of saving people’s lives helped to raise a large amount of donated funds and materials from other countries as well as domestic sources including small villages. Just in one month, more than 3.03 billion USD had been raised [16] and more than 10 million units of protective supplies had been sent to Wuhan [17].

Third, designated hospitals strived to establish humane administrative policies according to the state’s unified requirements [18]. All the designated hospitals in Wuhan offered various psychological counseling for patients and healthcare workers [19–21]; set up work shift intervals of 4–6 h in the wards instead of the regular 8 h [20]; and had experts on infection prevention consistently supervise healthcare workers’ anti-pandemic precautions and train healthcare workers on necessary prevention procedures [20]. In order to reduce the anxiety of the patients, nurses guided physical exercises for patients with milder symptoms in mobile cabin hospitals to distract them from the stress of uncertainty, and when necessary, volunteer professional psychologists provided bedside counseling for hospitalized patients [19]. Similar practices were also implemented in other regional designated hospitals, such as Guangzhou and Hunan [22, 23]. In addition, the local health commission and local residents strived to provide free accommodation, food, and transportation for frontline healthcare workers to guarantee their daily necessities [20, 21]. However, to date, less has been studied about the anti-pandemic nurses’ perception of hospital ethical climate, and how it affected the quality of care for COVID-19 patients.

Several studies [24–28] have indicated that nurses’ ethical sensitivity is associated both with ethical climate and care quality. According to some international literature [29–31], ethical sensitivity refers to recognizing the presence of ethical issues and making the right decisions by evaluating the patient’s situation and level of vulnerability, and the ethical consequences of any decisions made on behalf of the patient. Ethical issues occur when there are conflicts of values or interests, and moral judgment and choice are required. Ethical issues do not have solutions that are absolutely right or wrong [28]. For example, during the early stages of the COVID-19 outbreak, anti-pandemic nurses faced dilemmas between the professional duty to provide good care quality and the risk of self-infection due to the lack of sufficient protective supplies. They also found themselves in a social context where healthcare workers were portrayed as heroes, when in reality they could not save the life of every patient. Studies show that a high level of ethical sensitivity contributes to a high level of professionalism and increases care quality [26–28]. Considering that ethical sensitivity was also associated with ethical climate [24], and one study [32] indicated that ethical sensitivity played a mediating role between the ethical climate and nurses’ caring behavior, our study hypothesized that ethical sensitivity mediates the relationships between ethical climate and quality of care. However, to date, there have been no previous studies on anti-pandemic nurses’ ethical sensitivity, and its mediating role in the ethical climate and quality of care for COVID-19 patients.

Therefore, this study aims to (a) evaluate the strength of perceived hospital ethical climate, ethical sensitivity, and self-evaluated care quality among anti-pandemic nurses in China, (b) determine the relationships between the perceived hospital ethical climate and self-evaluated care quality for COVID-19 patients, and (c) explore the mediation effect of ethical sensitivity between perceived hospital ethical climate and self-evaluated care quality.

## Methods

### Study design

A cross-sectional study was conducted through an online survey among nurses who directly provided care for COVID-19 patients from December 2019 through April 2020 in Hubei, Hunan, and Sichuan province.

### Sample and setting

The target population was the anti-pandemic nurses who were directly providing care for COVID-19 patients in designated hospitals in Hubei, Hunan, and Sichuan provinces. Ten designated hospitals in six cities within these three provinces, namely Wuhan (the capital of Hubei province), Huanggang (a city in Hubei), Changsha (the capital of Hunan province), Chengdu (the capital of Sichuan province), Yibin, and Zigong (a city in Sichuan) were selected as study settings to recruit potential nurses. Potential participants were eligible for participating in this study if they were: (a) registered nurses, (b) worked in designated hospitals for at least two weeks (in order to adapt to the current work environment and be able to judge it), and (c) participated in direct COVID-19 patient care. Participants who withdrew early from the frontline clinical work due to suspected or confirmed COVID-19 infection were excluded.

### Data collection

Data were collected between March 2020 and April 2020 through Sojump, an online survey platform. We invited 10 volunteers (nursing managers) from 10 hospitals to dessiminate the study flyer to their WeChat groups (number of participants ranged from 27 to 122). Initially, a flyer was sent out to the targeted anti-pandemic nurses through the online chatting platform of WeChat. Those who were interested in this study could scan the QR code on WeChat to fill out the survey. Participants were given an electronic version of informed consent explaining the purpose, risk, and benefit of the study on the smartphone interface or the computer. Participants were required to read the informed consent at first; if they agreed to participate in the study they could click “agree” to start filling out the following survey. By submitting the online survey, they were provided with an opportunity for a random lottery prize valued from 1 to 2 RMB (0.144–0.288 USD). This had been set up in advance by the researcher and automatically performed by the Sojump online platform, and participants could easily accept the lottery payment by depositing it into their WeChat account. Long-in IDs were recorded automatically, and thus the person connected with each smartphone or computer ID could only submit the survey once. Filling out the survey required about 10 min; if the questionnaire was submitted within three minutes, it was deemed invalid. Participants could withdraw at any time. No personal identifiers were collected, and all the information was stored by the Sojump online company, which had a confidentiality contract with the research team.

### Instruments

#### Basic information sheet

The survey collected information on age, gender, marital status, educational background, qualification characteristics (including primary nurse, junior nurse, senior nurse, mainly through taking the corresponding professional qualification examination to obtain the corresponding professional qualification), professional work time (years), monthly income (RMB), and ethics training experiences.

#### Hospital ethical climate

This was measured using the Hospital Ethical Climate Survey (HECS), which measures how nurses perceive the ethical climate of their workplace. It was developed by Olson [8] in the United States in 1995, while the Chinese version was adapted by Wang et al. [33] in 2016. The Cronbach’s alpha of the Chinese version of HECS was 0.915 for the total scale [33]. It consists of 25 items, which are organized into five subscales, namely, relationship with peers (4 items), relationship with patients (4 items), relationship with managers (6 items), relationship with physicians (5 items), and relationship with hospital/organization (6 items). The responses are assessed on a 5-point Likert scale (ranging from 1 = “almost never true” to 5 = “almost always true”). A total score can be calculated, and the higher the score, the more positive the perception of hospital ethical climate.

#### Ethical sensitivity

This was measured using the Moral Sensitivity Questionnaire (MSQ), which was developed by Lützén [30] in 1994 to determine the ethical sensitivity of physicians and nurses who worked in healthcare workplaces. The Chinese Moral Sensitivity Questionnaire–revised version scale (MSQ-R-CV) used in this study was translated and localized by Huang et al. [34] in 2015. It consists of 9 items representing two factorssense of moral burden (4 items) and moral responsibility and strength (5 items). The Cronbach’s alpha coefficient was 0.820 for the total scale of MSQ-R-CV [34]. MSQ-R-CV is a self-administered questionnaire answered using a 6-point Likert scale (ranging from 1 = “I totally disagree” to 6 = “I totally agree”). The total score ranges from 9 to 54, and a higher total score reflects greater ethical sensitivity.

#### Self-evaluated care quality

This was measured using the Caring Behaviors Inventory-24 (CBI-24), a scale widely used in different countries to self-evaluate care quality by nurses. It was developed by Wolf et al. [35] in 1994 and modified by Wu et al. [36] in 2006. The CBI-24 scale we used was translated and adapted by Da et al. [37] in 2016. The Cronbach’s alpha coefficient was 0.959 for the total scale of the Chinese version of CBI-24 [37]. The inventory consists of 4 sub-scales of assurance (8 items), knowledge-skill (5 items), being respectful (6 items), and commitment (5 items). A 6-point Likert scale (ranging from 1 = “never” to 6 = “always”) was used for the responses. The higher the score, the better the quality of nursing care, based on self-evaluations by the nurses who provided it.

### Ethical considerations

This study was approved before data collection by the Institutional Review Board of Xiangya Nursing School of Central South University (Approval No. E202023). An electronic version of informed consent was obtained from the participants to ensure they fully understood the study’s purpose and procedures, and the risks and benefits of participating in it. Participation was voluntary and anonymous. All participants were assured of confidentiality and informed of their right to withdraw at any time. The data were only used for this study and would be destroyed at the end of the study.

### Data analysis

Data were exported directly from the online questionnaire and analyzed using SPSS 24.0. Frequencies and percentages were used to describe categorical variables, and means and standard deviations were used to describe the distributions of continuous variables. We compared the scores of different groups with *t*-test and one-way analysis of variance (one-way ANOVA) at a significance level of *p* < 0.05. We used Pearson’s correlations to test the relationship between the nurses’ perceived hospital ethical climate, their ethical sensitivity, and self-evaluated care quality.

Multiple linear regression analysis was conducted to examine whether ethical climate and ethical sensitivity were independently associated with care quality. A bootstrap test were performed for mediation analysis by SPSS with the PROCESS plug-in. The indirect, direct, and total effects of perceived hospital ethical climate on self-evaluated care quality via ethical sensitivity were determined. Following the procedure recommended by Hayes [38], we chose Model 4. According to our hypothesis, self-evaluated care quality was the dependent variable, perceived hospital ethical climate was the independent variable, and ethical sensitivity was a mediator. Concurrent covariates were gender, education, working years as a nurse, and ethics training experience. The level of significance was set at 0.05 (two-sided). An effect was considered significant if its 95% bootstrap confidence interval from 5,000 bootstrap samples did not include zero. Finally, we ran a boostrap analysis to calculate the effects that (a) perceived hospital ethical climate had on moral sensitivity, (b) ethical sensitivity had on self-evaluated care quality, and (c) perceived hospital ethical climate had on self-evaluated care quality.

## Results

### Participant characteristics

We collected a total of 439 questionnaires, of which 399 with completed information were used for analysis. Among the nurses with complete responses, 219 came to Wuhan to aid the anti-pandemic health services: 118 from Sichuan, 22 from Hunan, 16 from Chongqing, 15 from Guizhou, 12 from Jiangsu, 10 from Shanxi, 9 from Henan, and 17 from other areas. Among the other responding nurses, 180 were working in local designated hospitals: 22 in Hubei, 137 in Sichuan, and 21 in Hunan. The participants’ average age was 31.53 ± 6.269 years. The majority (84.5%) were female, married (65.2%), and had earned bachelor’s degrees or above (78.2%). More than two-thirds of participants have been working as nurses for 6–15 years (66.5%). Almost half of the participants had a monthly income of more than 6000.0 RMB (US $846.10). Nearly three quarters had received ethics training (76.2%) (Table 1).

**Table 1:** Characteristics of participants in the study (n = 399)

Characteristics	Categories	n	%
Gender	Male	62	15.5
	Female	337	84.5
Marital status	Single	107	26.8
	Married	260	65.2
	Divorced	26	6.5
	Widowed	6	1.5
Education	Certificate (technical school)	7	1.8
	Associate degree	80	20.1
	Bachelor’s degree	293	73.4
	Master’s degree	19	4.8
Qualification	Primary nurse	260	65.1
	Junior nurse	112	28.1
	Senior nurse	27	6.8
Working years as a nurse (years)	≤ 5	60	15.0
	6–10	179	44.9
	11–15	86	21.6
	16–20	35	8.8
	> 20	39	9.8
Monthly income (RMB)	≤ 3999.0 (≤ US$612.8)	42	10.5
	4000.0–5999.0 (US$612.8–US$919.2)	162	40.6
	6000.0–7999.0 (US$919.2–US$1225.6)	110	27.6
	8000.0–9999.0 (US$1225.6–US$1532)	59	14.8
	≥ 10,000.0 (≥ US$1532)	26	6.5
Ethics training experience	Yes	304	76.2
	No	95	23.8

### The status of hospital ethical climate, ethical sensitivity, and self-evaluated care quality

The average scores for HECS (4.43 ± 0.577), MSQ-R-CV (45.00 ± 7.085), and CBI-24 (5.34 ± 0.661) were relatively high (Table 2).

**Table 2:** Means and SDs for HECS, MSQ-R-CV, and CBI-24 sub-scale scores (n = 399)

Scales and sub-scales	Mean	SD	Range
HECS	4.43	0.577	1–5
HECS peers HECS patients	4.48 4.46	0.617 0.592	1–5 1–5
HECS managers	4.48	0.627	1–5
HECS physicians	4.32	0.620	1–5
HECS hospital	4.41	0.615	1–5
MSQ-R-CV	45.00	7.085	9–54
Moral responsibility and strength	26.23	3.848	5–30
Sense of moral burden	18.77	4.163	4–24
CBI-24	5.35	0.661	1–6
Assurance	5.41	0.782	1–6
Being respectful	5.34	0.710	1–6
Knowledge-skill Commitment	5.42 5.23	0.733 0.774	1–6 1–6

HECS: Hospital Ethical Climate Survey; MSQ-R-CV: Moral Sensitivity Questionnaire- Revised Version into Chinese; CBI-24: Caring Behaviors Inventory 24

SD: Standard Deviation

### The correlation between hospital ethical climate, ethical sensitivity, and self-evaluated care quality

The Pearson correlation test results showed a significant correlation between hospital ethical climate and self-evaluated care quality (r = 0.801, *p* < 0.01). Participants with a higher MSQ-R-CV score also had higher scores on CBI-24 (r = 0.604, *p* < 0.01). There was a positive significant relationship between hospital ethical climate and ethical sensitivity (r = 0.680; *p* < 0.01).

Multiple linear regression analysis showed that hospital ethical climate (β = 0.710, *p* < 0.01) and ethical sensitivity (β = 0.114, *p* < 0.001) were independently associated with self-evaluated care quality (Table 3).

**Table 3:** The associated factors of self-evaluated care quality by the multiple linear regression model (n = 399)

Independent variable	B	SE	β	t
Gender	0.055	0.084	0.020	0.665
Education	0.080	0.056	0.043	1.425
Working years as a nurse	– 0.048	0.026	– 0.055	– 1.824
Ethics training experience	– 0.050	0.071	– 0.021	– 0.705
Hospital ethical climate	1.231	0.073	0.710	16.974***
Ethical sensitivity	0.016	0.006	0.114	2.792**

Adjusted R^2^ = 0.648

**p < 0.01; *** *p* < 0.001

### Mediation analysis of ethical sensitivity on self-evaluated care quality

**Fig. 1:**
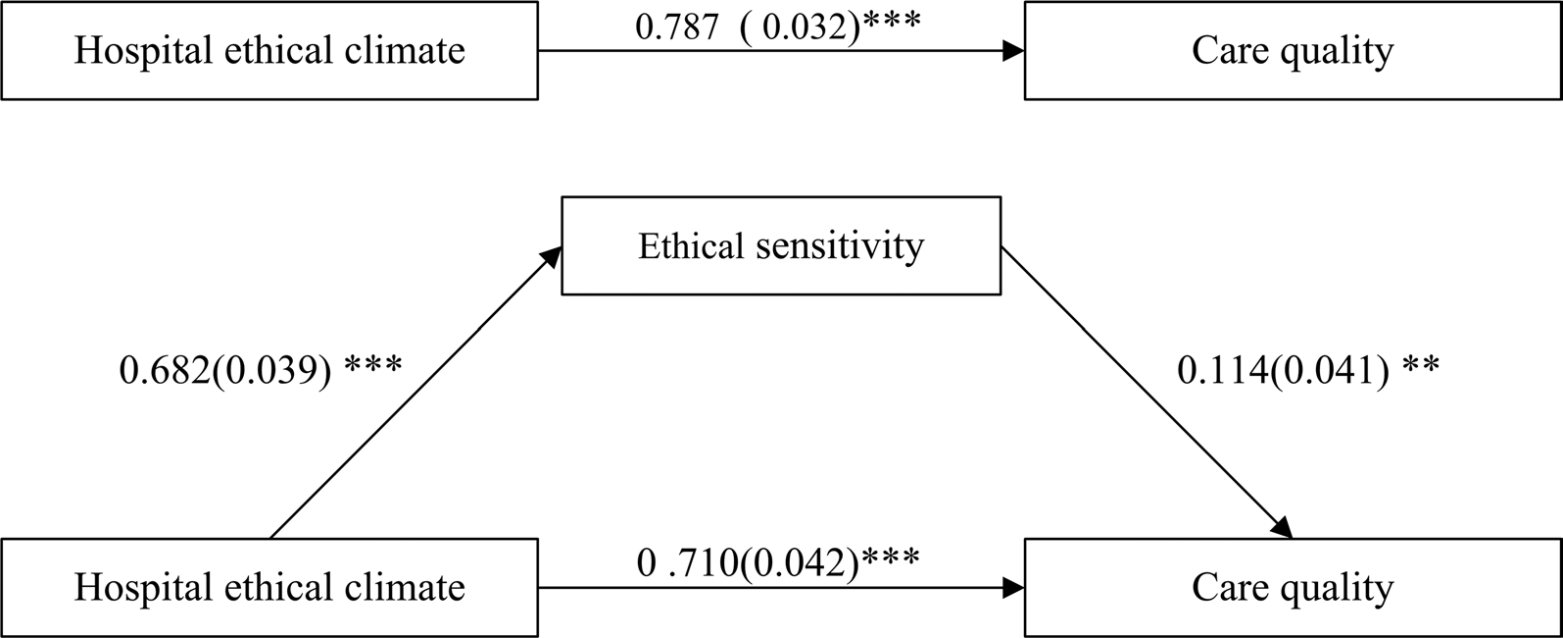
Mediation model linking hospital ethical climate with self-evaluated care quality through ethical. **p < 0.01; ***p < 0.001

Table 4 shows pathway estimates and 95% CIs for the mediation model. The direct and indirect effects’ 95% CIs did not include zero; therefore, the mediating role of ethical sensitivity was significant in the self-evaluated care quality model. As shown in Table 4 and Fig. 1, when ethical sensitivity was included in the model, the previously significant path between hospital ethical climate and self-evaluated care quality was still significant but decreased (from 0.787 to 0.710, *p* < 0.001). Overall, these findings indicated a consistent pattern in which ethical sensitivity partly mediated the association between hospital ethical climate and self-evaluated care quality.

**Table 4:** Effects of the mediator on self-evaluated care quality: mediation model (n = 399)

Effect	Product of coefficients		95% CI	
	Point estimate	SE	LL CI	UL CI
Total effect (c) Direct effect (c′) Indirect effect (a·b)	0.787 0.710 0.078	0.032 0.042 0.036	0.725 0.628 0.002	0.849 0.792 0.145

CI: Confidence Interval; LL: Lower Limit; UL: Upper Limit

## Discussion

This study targeted Chinese anti-pandemic nurses who directly cared for COVID-19 patients. It showed that these nurses reported high average scores for hospital ethical climate, ethical sensitivity, and self-evaluated quality of care for COVID-19 patients. This study also found that among anti-pandemic nurses, the perceived strength of the hospital ethical climate was associated with high self-evaluated care quality directly, as well as indirectly through the mediation effect of ethical sensitivity.

Anti-pandemic nurses in our study perceived a high degree of strength in the hospital ethical climate. The average scores for this measurement were higher than those reported by nurses using the same study instrument in acute-care hospitals in Canada in 2009 [39]; in care settings for older people in Finland in 2015 [40]; and in cancer care settings in Greece and Cyprus in 2019 [41] —although the difference might be due to differences in working settings, cultural backgrounds, and situational contexts. An organization’s values and goals could reflect the strength of the ethical climate in that organization [42], which provides the context in which ethical behavior and decision-making occurs. Thus, if nurses have a deep understanding of the hospital ethical climate, they can judge and adjust their behaviors to be consistent with the hospital’s values. During the COVID-19 outbreak, the rapidly evolving pandemic threatened the health and lives of the public. In that critical period, making people’s lives and health the priority and making all-out efforts to prevent and control the contagion was society’s common goal [43, 44]. China coped with the rapid spread of the COVID-19 pandemic by building two temporary hospitals in two weeks (Leishenshan and Huoshenshan), and transforming exhibition halls, stadiums, hotels and college dormitories into places to isolate the patients diagnosed with mild symptoms [44]. Even though nurses were at risk of infection, they experienced a professional obligation not to withdraw from participating in the anti-pandemic efforts. However, we should note that these data were collected from March through April 2020, when the nurses had only worked in the isolated wards for a couple of months. Some negative factors such as burnout that affect nurses’ perceptions of hospital ethical climate were likely not yet obvious or severe [45].

In addition, although nurses reported both high scores on HECS physicians and managers/peers, our study also showed that nurses perceived more positive relationships with managers and peers than with physicians, which was consistent with previous studies [33, 39, 40]. On the one hand, the nursing profession traditionally requires obedience to hospital managers and a high level of cooperation with peers. On the other hand, the long-standing affiliation in seniority hierarchies between physicians and nurses may have been a cause of the relatively low score on relationships with physicians as perceived by nurses [40]. During the pandemic, different work shifts and tasks for physicians and for nurses might further reduce their contact. For example, the general isolation hospital wards or mobile cabin hospital wards were mainly under the charge of nurses, and the physicians had a more consultative role. Therefore, strengthening physician-nurse cooperation on empirical clinical work is crucial to improve the hospital ethical climate.

Our study indicated the anti-pandemic nurses demonstrated a higher level of ethical sensitivity than those reported by nurses working in the psychiatric department in Sweden in 2009 [24, 46], and nurses working in tertiary and secondary hospitals in China in 2018 and 2014 [47, 48]. According to Huang et al. [34], ethical sensitivity includes moral responsibility and strength, and moral burden dimensions. As the positive dimension of ethical sensitivity, moral responsibility and strength play an important role in nurses’ cognition and judgment when facing ethical issues in daily work. In contrast, moral burden is considered to be the negative dimension, evoked by a problem or situation. In our study, we have strong reason to believe the anti-pandemic nurses’ moral responsibility and strength, and moral burden were both higher than in previous studies [34, 47]. On the one hand, anti-pandemic nurses volunteered to go to the frontline and thus could have had high professional commitment and compassion [15, 49, 50], which could explain our results showing high moral responsibility and strength. But anti-pandemic nurses could also easily encounter conditions that could lead to a high level of moral burden, such as the challenges of excessive psychological pressure, the lack of experience in dealing with the new disease, insufficient supplies of protective gear, overload of facilities, and the high risk of infection [49, 51, 52]. The high ethical sensitivity demonstrated by nurses in this study suggests that they were shaped by the institutional mission of the anti-pandemic hospitals, and were actively trying to address the ethical burdens they faced on the frontlines. Future research should examine how supportive services—such as the psychological counseling, aid for daily necessities, and short work shifts available to the nurses in this study—can continue to be improved during responses to public health emergencies, to help alleviate the moral burden dimension of ethical sensitivity.

The anti-pandemic nurses reported a high level of self-evaluated care quality in this study, which was against the general assumption that care quality could not be guaranteed due to the high requirements for occupational precautions. A recent qualitative study showed that anti-pandemic nurses felt proud that they had the opportunity to serve and fulfill their professional duties during the COVID-19 outbreak in China [53]. The feeling of being needed by their country and people encouraged them to join the anti-pandemic task without hesitation [53]. Because of the national call to respond to COVID-19, nurses in studies and news reports appeared to experience the pandemic response not only as a professional duty but also ethically imperative. Notably, the subscale titled “commitment” showed a relatively lower score than other sub-scales on the self-evaluated care quality scores, and the item “spending time with the patient” gained the lowest score. These findings may be a result of the requirements for nurses to distance themselves from patients and take additional occupational precautions to prevent the spread of infection. Other research shows that wearing medical protective masks, disposable protective suits, and disposable latex gloves during the pandemic response reduced the sensory stimulation of anti-pandemic nurses and even caused sensory deprivation [54], which could have also affected communication and connection with patients. However, intrinsic motivation of nurses helped them to make great efforts to adhere to professional values [15, 55], which might have been a cause of the overall high level of self-evaluated care quality.

Similar to previous studies [56, 57], our study revealed that the perceived hospital ethical climate, ethical sensitivity, and self-evaluated care quality were positively correlated with each other and that ethical sensitivity partly mediated the relationship between hospital ethical climate and self-evaluated care quality. Nurses played a crucial role in the treatment and care of COVID-19 patients, and ethical sensitivity was essential for nurses to provide high-quality care. A high ethical sensitivity level could help nurses feel confident in justifying ethical decisions, feel prepared to deal with ethical issues, and feel confident in fulfilling professional responsibilities [58, 59]. Some studies [33, 34] have translated and localized the hospital ethical climate and ethical sensitivity survey tools for Chinese culture. Therefore, future studies can develop training programs in China and other countries to improve the hospital ethical climate and ethical sensitivity of healthcare workers, especially for public health nurses, to equip them with the ethical capacity to perform well during public health emergencies.

Interestingly, some variables that were not shown to be significantly associated with quality of care, namely education background, working years as a nurse, and ethics training experience. There might be some reasons. First, the proportion of nurses with a bachelor’s degree and above in this study (73.4%) was much higher than that in whole China [60]. Second, most of these nurses who were selected and sent out to Wuhan were excellent ones, and thus, the working years might not be associated with quality of care. Third, ethical trainings for nurses were not comprehensive and intensive for all nurses in China, even some of them reported receiving some kind of ethical training, the quality was unsure [61]. Therefore, our study did not show the association between ethical training and quality of care.

There are several limitations to this study. First, the self-reported data may lead to social desirability bias. Given that anti-pandemic nurses were rightfully perceived as heroes by the public, they may have answered the questions in a more positive or socially desirable way. We used an anonymous surveyto minimize the social desirability bias. Second, nonrandomized sampling methods were used to select the study settings and participants due to the urgent situation, which may create a selection bias. Those who were interested in this study might not be represtative of the who anti-pandemic nurses. Third, the sample was confined to several regions of Mainland China. Due to the cultural context and the condition of pandemic differences between different regions and countries, the generalization of the findings to other culture different areas or counties was limited. Last not the least, the data were cross-sectional, which limited the ability to make causal inferences. Nevertheless, we believe that our data could be considered as a fair reflection of hospital ethical climate, self-evaluated care quality, and ethical sensitivity of Chinese anti-pandemic nurses regarding taking care of COVID-19 patients, given that our sample consisting nurses from both general and the epicenter of the pandemic (Wuhan) designated hospitals.

## Conclusions

This study targeted Chinese anti-pandemic nurses who directly cared for COVID-19 patients and provided essential insights into the association of hospital ethical climate with nurses’ self-reported care quality during the early stage of the COVID-19 pandemic. Overall results of this study reveal that these nurses had positive perceptions of hospital ethical climate, ethical sensitivity, and self-evaluated quality of care for COVID-19 patients. This study also identified that the perceived high level of hospital ethical climates was associated with high self-reported care quality directly, and also indirectly through the mediation effect of ethical sensitivity. The study suggests that improving hospital ethical climate and ethical sensitivity might improve care quality in clinical settings.

## Data Availability

The datasets used and/or analysed during the current study are available from the corresponding author on reasonable request.

## Abbreviations

CI: Confidence interval
HECS: Hospital ethical climate survey
MSQ-R-CV: Moral sensitivity questionnaire-revised version into Chinese
CBI-24: Caring behaviors inventory 24
SD: Standard deviation
LL: Lower limit
UL: Upper limit
